# *Pseudomonas aeruginosa* Bloodstream Infection, Resistance, and Mortality: Do Solid Organ Transplant Recipients Do Better or Worse?

**DOI:** 10.3390/antibiotics12020380

**Published:** 2023-02-13

**Authors:** Sabina Herrera, Laura Morata, Abiu Sempere, Miguel Verdejo, Ana Del Rio, Jose Antonio Martínez, Guillermo Cuervo, Marta Hernández-Meneses, Mariana Chumbita, Cristina Pitart, Pedro Puerta, Patricia Monzó, Carles Lopera, Francesco Aiello, Scarleth Mendoza, Carolina Garcia-Vidal, Alex Soriano, Marta Bodro

**Affiliations:** 1 Department of Infectious Diseases, Hospital Clínic, 08036 Barcelona, Spain; 2 Department of Microbiology, Hospital Clínic, 08036 Barcelona, Spain; 3 Centro de Investigación Biomedical en Red en Enfermedades Infecciosas CIBERINFEC, 28029 Madrid, Spain

**Keywords:** *Pseudomonas aeruginosa*, bloodstream infection, solid organ transplant, carbapenem-resistant, multi-drug resistance

## Abstract

Background: The prevalence of antimicrobial resistance of *Pseudomonas aeruginosa* (*P. aeruginosa*) in solid organ transplant (SOT) recipients is higher than that of the general population. However, the literature supporting this statement is scarce. Identifying patients at risk of carbapenem resistance (CR) is of great importance, as CR strains more often receive inappropriate empiric antibiotic therapy, which is independently associated with mortality in bloodstream infections (BSIs). Methods: We prospectively recorded data from all consecutive BSIs from January 1991 to July 2019 using a routine purpose-designed surveillance database. The following variables were included: age, sex, type of transplant, use of vascular and urinary catheters, presence of neutropenia, period of diagnosis, treatment with steroids, origin of BSI, source of bacteremia, septic shock, ICU admission, mechanical ventilation, previous antibiotic treatment, treatment of bacteremia, and 30-day all-cause mortality. Results: We identified 2057 episodes of *P. aeruginosa* BSI. Of these, 265 (13%) episodes corresponded to SOT recipients (130 kidney transplants, 105 liver, 9 hearts, and 21 kidney–pancreas). Hematologic malignancy [OR 2.71 (95% CI 1.33–5.51), *p* = 0.006] and prior carbapenem therapy [OR 2.37 (95% CI 1.46–3.86), *p* < 0.001] were associated with a higher risk of having a CR *P. aeruginosa* BSI. Age [OR 1.03 (95% CI 1.02–1.04) *p* < 0.001], urinary catheter [OR 2.05 (95% CI 0.37–3.06), *p* < 0.001], shock at onset [OR 6.57 (95% CI 4.54–9.51) *p* < 0.001], high-risk source [OR 4.96 (95% CI 3.32–7.43) *p* < 0.001], and bacteremia caused by CR strains [OR 1.53 (95% CI 1.01–2.29) *p* = 0.036] were associated with increased mortality. Correct empirical therapy was protective [OR 0.52 (95% CI 0.35–0.75) *p* = 0.001]. Mortality at 30 days was higher in non-SOT patients (21% vs. 13%, *p* = 0.002). SOT was not associated with a higher risk of having a CR *P. aeruginosa* BSI or higher mortality. Conclusions: In our cohort of 2057 patients with *P. aeruginosa* BSIs, hematologic malignancies and previous carbapenem therapy were independently associated with a risk of presenting CR *P. aeruginosa* BSI. Age, urinary catheter, high-risk source, bacteremia caused by carbapenem-resistant strains, and severity of the infection were independently associated with mortality, whereas correct empirical therapy was a protective factor. An increasing trend in the resistance of *P. aeruginosa* was found, with >30% of the isolates being resistant to carbapenems in the last period. SOT was not associated with a higher risk of carbapenem-resistant BSIs or higher mortality.

## 1. Introduction

Bacterial infections constitute a major source of morbidity and mortality in solid organ transplant (SOT) recipients. Among bacterial infections, bloodstream infections (BSIs) occur in over one-third of SOT recipients and are associated with high mortality [1]. Gram-negative bacilli (GNB) have become the leading agents of bloodstream infections (BSIs) in SOT recipients [2,3], probably as a consequence of a reduction in catheter-related BSIs [4]. *Pseudomonas aeruginosa* (*P. aeruginosa*) accounts for up to 8% to 15% of all BSIs in SOTs [5,6].

In line with the worldwide spread of the so-called high-risk clones of multidrug-resistant or extensively drug-resistant (MDR/XDR) *Pseudomonas aeruginosa* [7], the prevalence of antimicrobial resistance of *P. aeruginosa* in SOT recipients is higher than that of the general population [6,8]. This is not surprising, as SOT patientspresent several of the risk factors for MDR *P. aeruginosa* BSI. SOT patients are recurrently exposed to antibiotic treatment, hospital environments, invasive procedures (catheters), and immunosuppressive therapies [9]. Identifying patients at risk of MDR/CR is of great importance, as CR strains more often receive inappropriate empiric antibiotic therapy, which is independently associated with mortality in BSIs [10,11].

The aim of our study is to describe the epidemiology, clinical characteristics, resistance patterns, and clinical outcomes of *P. aeruginosa* BSIs in a large cohort of bloodstream infections and analyze whether transplantation could be considered a risk factor for multidrug-resistant infections and/or mortality. 

## 2. Methods

### 2.1. Setting

We conducted a retrospective study at a tertiary university referral hospital with an active solid organ transplant program in Barcelona, Spain. We prospectively recorded data from all consecutive BSIs from January 1991 to July 2019 using a routine purpose-designed surveillance database.

In addition to microbial species and antibiotic susceptibilities, the following variables were also included in the database: age, sex, type of transplant, use of vascular and urinary catheters, presence of neutropenia, treatment with steroids, origin of BSI, focus of bacteremia, outcomes in terms of admission to ICU, mechanical ventilation, septic shock, and mortality at 30 days. Previous antibiotic treatment, treatment of bacteremia, and appropriateness of empirical treatment were also recorded. 

### 2.2. Definitions

(1) Bloodstream infection (BSI): Bloodstream infection was defined as a bacterial infection identified via blood culture. The bacteremia source was determined on the basis of clinical criteria and isolation from a clinically significant site of infection of the same organism found in the blood isolate, on the basis of species identification and antibiotic susceptibility results. Central line-associated BSI was defined as positive blood culture in the presence of a central line, with the source of BSI documented as line-associated by the treating clinical team. Catheter-related bacteremia was documented when the blood isolate was cultured from the catheter tip (≥10^3^ cfu/mL).
(2) Bacteremia was considered to be primary or of unknown source in patients in whom no source of bacteremia was identified.
(3) Carbapenem resistance (CR) was defined if the isolate tested resistant to at least one of the carbapenem antibiotics (meropenem, doripenem, or imipenem).
(4) Septic shock: Sepsis episodes requiring the use of vasopressors due to persistent hypotension despite fluid therapy with a causal and temporal relationship with the BSI episode [12].
(5) Bacteremia was considered to be hospital-acquired, healthcare-related or community-acquired, as described elsewhere [13]. Empirical antibiotic therapy was considered appropriate when the patient received a proper dosage by an adequate route of at least one in vitro active antimicrobial agent within 24 h after blood cultures were obtained and before antibiotic susceptibility results were reported.
(6) Prior antibiotic therapy was defined as the receipt of any systemic antibiotic for ≥48 h in the previous month.
(7) Persistent bacteremia: Two or more positive blood cultures obtained on different calendar days with isolation of the same microorganism during the same infectious episode.
(8) Neutropenia: Less than 500 neutrophils per microliter of blood.

Patients were followed up for 30 days after the onset of bacteremia. 

### 2.3. Microbiological Procedures

Samples for blood cultures were inoculated into aerobic and anaerobic vials and processed by the Bactec 9240 System (Becton–Dickinson, Block Scientific Inc Bellport NY 11713 United States), with an incubation period of 5 days. Antimicrobial susceptibility testing was performed using a microdilution system or E-test. Susceptibility to antimicrobials was established according to the Clinical and Laboratory Standards Institute (CLSI) breakpoints until mid-2011 and to the current European Committee on Antimicrobial Susceptibility Testing (EUCAST) since that date.

### 2.4. Statistical Analysis

All calculations were performed with the SPSS statistical package (version 18.0; SPSS, Chicago, IL, USA). Categorical variables were compared using the *χ^2^* or Fischer exact test as appropriate. Continuous variables were compared using either the Student *t*-test or non-parametric tests depending on the homogeneity of the variable. We used the Kaplan-Meier method to perform survival curves. We assessed the impact of age, sex, use of vascular and urinary catheters, presence of neutropenia, treatment with steroids, origin of BSI, focus of bacteremia, septic shock, and appropriateness of empirical treatment on the risk of having carbapenem-resistant (CR) *P. aeruginosa* BSI, and attributed mortality using a logistic regression model to calculate odds ratios (OR) and 95% confidence intervals (CIs). All statistical tests were two-tailed, and the threshold of statistical significance was set at *p* < 0.05.

## 3. Results

During the study period, we identified 2057 episodes of *P. aeruginosa* BSI (Table 1). Sixty four percent corresponded to men, and the median age of the cohort was 63 years (SD 19). Notably, 18% had diabetes mellitus, 10% chronic pulmonary obstructive disease, 15% ischemic cardiomyopathy, 22% hematologic malignancy, 6% liver cirrhosis, and 6% HIV. Most of the BSIs were hospital-acquired (85%), 64% of the patients carried venous catheters, 33% carried urinary catheters, and 15% had neutropenia. The main source of bacteremia was catheter-related in 31% of patients, followed by primary bacteremia in 22%, urinary tract infection in 15%, respiratory in 15%, and abdominal infection in 14%. 

Almost one in three patients (29%) received an inappropriate empirical antibiotic therapy, 19% had septic shock due to the BSI, 15% required ICU admission, and 10% mechanical ventilation. Moreover, 13% had persistent BSIs and 20% died within 30 days. 

In the logistic regression model (Table 2), hematologic malignancy [OR 2.71 (95% CI 1.33–5.51), *p* = 0.006] and prior carbapenem therapy [OR 2.37 (95% CI 1.46–3.86), *p* <0.001] were associated with a higher risk of having a CR PAE BSI. 

Table 3 shows the logistic regression model of variables evaluated as predictive factors of mortality in patients presenting with *P. aeruginosa* BSIs. The presence of septic shock [OR 6 (95% CI 4–8.5), *p* < 0.001], high-risk source (all sources except urinary tract infection and catheter-related infection) [OR 4.96 (95% CI 3.32–7.43) *p* < 0.001], use of urinary catheter [OR 2.05 (95% CI 0.37–3.06), *p* <0.001], bacteremia caused by CR strains [OR 1.53 (95% CI 1.01–2.29) *p* = 0.036], and age [OR 1.03 (95% CI 1.02–1.04) *p* <0.001] were associated with increased mortality. Appropriate empirical therapy was protective [OR 0.5 (95% CI 0.3–0.7) *p* < 0.001].

**Figure 1:**
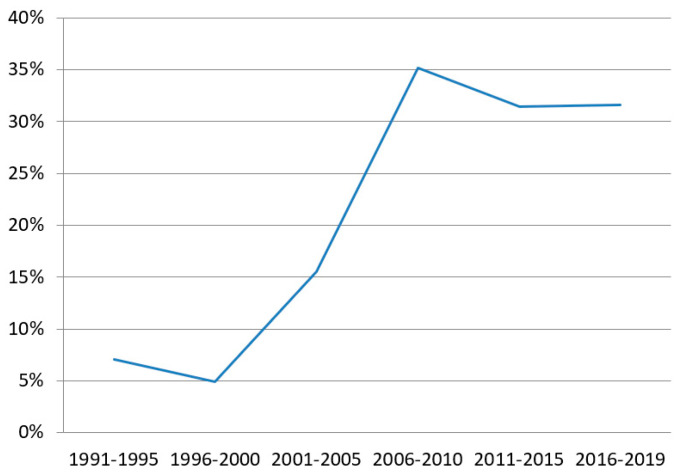
Percentage of carbapenem-resistant *Pseudomonas aeruginosa* in bloodstream infections though time.

Four hundred seventy-five episodes (24%) were carbapenem-resistant. Trends in *P. aeruginosa* resistant to carbapenems are depicted in Figure 1. In the period from 1991 to 1995, 7% of the isolates were resistant to carbapenems. This percentage was doubled in the period from 2001 to 2005 (15.5%) and continued to increase in the following periods, with up to 31.6% of the isolates being CR in the last period.

### Solid Organ Transplant

Of the 2057 episodes of *P. aeruginosa* BSI, 265 (13%) episodes corresponded to solid organ transplant recipients (130 kidney transplants, 105 liver transplants, 9 hearts transplants, and 21 kidney–pancreas transplants) Table 4. Compared to the non-SOT population, the SOT patients were significantly younger, had significantly higher rates of diabetes mellitus, were significantly less neutropenic, and carried fewer urinary catheters. Most of the BSIs were of urinary origin (26.6%), mainly lower-tract urinary tract infections, followed by catheter-related BSIs (25%) and primary bacteremia (16%). Almost one in five (18%) presented with septic shock. Twenty-eight percent of patients received an inadequate empirical antibiotic therapy similar to the non-SOT population. In terms of outcomes, the non-SOT population required significantly more ICU admissions and mechanical ventilation compared to the SOT group. Twenty-one percent of the patients died within 30 days in the non-SOT group and thirteen percent died in the SOT group (*p* = 0.002). 

Table 5 shows the logistic regression model of variables evaluated as predictive factors of carbapenem-resistant *P. aeruginosa* bloodstream infections in SOT patients. Urinary catheter [OR 1.98 (95% CI 1.1–3.69), *p* = 0.031], venous catheter [OR 2.05 (95% CI 1.8–4.25), *p* = 0.013], and prior carbapenem therapy [OR 4.49 (95% CI 2.10–9.95), *p* <0.001] were associated with a higher risk of having a CR *P. aeruginosa* BSI.

## 4. Discussion

In our large cohort of bloodstream infections caused by *Pseudomonas aeruginosa,* we found that hematologic malignancies and previous carbapenem therapy were independently associated with a risk of presenting CR *P. aeruginosa* BSIs. Older age, presence of urinary catheter, high-risk source, bacteremia caused by carbapenem-resistant strains, and severity of the infection were independently associated with mortality, whereas correct empirical therapy was a protective factor. Thirteen percent of the BSIs occurred in solid organ transplant recipients. Transplantation was not associated with a higher risk of antibiotic resistance or mortality. To our knowledge, this is the largest *P. aeruginosa* BSI reported in SOT recipients to the date.

In our series, we found hematologic malignancies and previous carbapenem therapy to be the only risk factors associated with MDR BSIs. These variables may act as a surrogate marker for other variables that might increase the probability of colonization by drug-resistant strains, such as antibiotic exposure and health care exposure, as our study and others have found. However, we did not find transplantation to be a risk factor for CR *P. aeruginosa* BSIs. Transplant patients, like patients with hematological malignancies, have classically been considered to be at high risk for colonization and infections caused by CR Gram-negative bacteria. The hypothesis behind this statement is that their frequent contact with the healthcare system, and especially the exposure to multiple antibiotics that affect the composition of the intestinal microbiota, make the patient susceptible to MDR pathogen colonization [14]. Colonization of the intestinal tract results in major risk of suffering from invasive infections, including bacteremia. Bacteremia is less frequent in SOT compared to neutropenic patients, but is still a life-threatening complication. In our group of SOT patients, we found that those carrying urinary catheters, venous catheters, and those with prior carbapenem treatment were at higher risk of having a CR strain. Studies specifically analyzing *P. aeruginosa* BSIs in SOT recipients found prior transplantation, nosocomial acquisition, ICU admission, and septic shock at onset as risk factors [8,15] for MDR *P. aeruginosa* BSIs. 

In our center, the trends in CR have shown a gradual increase throughout the years, showing that more than 30% of the isolates in the last decade are resistant to carbapenems. Other studies with time monitoring of carbapenem resistance in *P. aeruginosa* BSIs have also shown increasing resistance, with more than 25% of the isolates being resistant [16,17]. In SOTs, Johnson et al. [8] found that single-drug non-susceptibility and MDR *P. aeruginosa* were greater in transplant recipients compared with non-transplant patients, with close to >30% resistance to imipenem in 2005. Surprisingly, Oriol et al. [2] found that the rate of MDR *P. aeruginosa* remained unchanged over time in their 10-year study of BSIs in SOTs following the first year post-transplant. Their study period spanned the years 2006 to 2016, where we also found no increase in the rate of CR *P. aeruginosa*, probably because of the implementation of infection control programs. 

Similar to ours, several studies have demonstrated that patients with MDR or XDR *P. aeruginosa* BSIs have worse outcomes than patients with non-MDR *P. aeruginosa* BSIs [6,18,19,20,21,22]. However, this fact seems more related to a higher frequency of inadequate empirical antibiotic therapy than to the resistance of the *P. aeruginosa* itself. In our cohort, we found that one-third of the patients received inadequate empirical antibiotic treatment. Following the current protocols, where a carbapenem is given as empirical treatment in Gram-negative bacilli BSIs, we fail to identify and treat promptly one in three patients with *P. aeruginosa* BSIs. This is of high importance given that inadequate empirical treatment is associated with a higher mortality rate, as our study and previous studies have repeatedly demonstrated [10,23,24]. Accordingly, it is necessary to consider new beta-lactams with activity against MDR *P. aeruginosa* (ceftazidime-avibactam, ceftolozane-tazobactam or cefidercol) as empirical therapy in patients at risk.

In addition to inadequate empirical antibiotics, age, urinary catheter, high-risk source, and severity of the infection were also associated with higher mortality. High-risk source, such as abdominal or pulmonary source, was found to be a risk factor for 30-day mortality, in line with other studies that found worse outcomes in bacteremia of respiratory origins compared to other sources [25,26,27]. Factors reflecting the severity of the infection such as septic shock were logically associated with mortality. Tumbarello et al. [24] found that those with septic shock had a higher 21-day mortality risk. 

Surprisingly, SOT was not associated with a higher risk of mortality. There were baseline differences in the groups that could explain this fact, such as higher percentage of patients with hematological malignancies in the non-SOT group, higher use of urinary catheters, and more pulmonary sources. On the other hand, there were no baseline differences in terms of septic shock or use of inadequate empirical therapy. Most of the literature concerning *P. aeruginosa* BSIs in SOTs is reflected in studies that analyze BSIs retrospectively, including other Gram-negative bacilli, but not specifically *P. aeruginosa*. Small numbers generally preclude these studies from further analyzing specific risk factors in the SOT population. In the study by Johnson et al. [8], onset of BSI while in the ICU was the only independent predictor of 28-day in-hospital mortality for SOT patients with *P. aeruginosa* BSIs. However, in the study by Bodro [6] et al., BSIs due to XDR *P. aeruginosa*, presence of co-infection, catheter source, and primary source were found to be independent risk factors for mortality. Recently, Eichenberger et al. [28] broke the stigma that SOT patients have poorer outcomes, finding that SOT recipients presenting with Gram-negative BSIs do not experience higher rates of septic shock, respiratory failure, or mortality compared to non-SOT recipients. It has been hypothesized that a greater number of immunosuppressive medications may in fact be associated with improved outcomes in Gram-negative BSIs [29]. Eichenberger et al. found that some cytokines and chemokines were significantly lower in the SOT population, implying that the lower inflammatory response could justify the lower mortality rates compared to patients not receiving immunosuppressive therapies [28]. However, further studies are required to confirm these results. 

Our study has several limitations. Because it is a single-center study, our findings may be attributable to institution-specific variables and may not reflect the epidemiology of different centers and geographic areas. Our center does not perform lung transplants, a procedure where patients have a high burden of *P. aeruginosa*, underestimating the prevalence of *P. aeruginosa* itself, and more specifically MDR or XDR *P. aeruginosa*. There were baseline differences in the SOT and non-SOT groups. Additionally, we did not have information regarding previous patient colonization. Specific genotypes and serotypes [30,31] that have been associated with higher mortality were not assessed. 

In summary, in our cohort of more than 2000 patients with *P. aeruginosa* BSIs, hematologic malignancies, and previous carbapenem therapy were independently associated with a risk of presenting CR *P. aeruginosa* BSIs. Age, urinary catheter, high-risk source, bacteremia caused by CR strains, and severity of the infection were independently associated with mortality, whereas correct empirical therapy was a protective factor. We found an increasing trend in the resistance of *P. aeruginosa*, with >30% of the isolates being resistant to carbapenems in the last decade. Solid organ transplantation was not associated with a higher risk of CR *P. aeruginosa* BSIs, or with a higher risk of mortality. Efforts should be focused on identifying patients at risk of CR *P. aeruginosa* to offer adequate empirical treatment in a prompt manner.

## Figures and Tables

**Table 1:** Demographics and clinical characteristics of the cases with *P. aeruginosa* BSI and their outcomes.

	Total n = 2057
Men	64%
Age, median (SD)	63 (SD19)
Comorbidities	
Diabetes mellitus	18%
COPD	10%
Ischemic cardiopathy	15%
Hematologic malignancy	22%
Liver cirrhosis	6%
HIV	6%
Risk factors	
Urinary catheter	33%
Venous catheter	64%
Neutropenia	15%
Hospital-acquired	85%
Community-acquired	15%
Source	
Catheter-related	31%
Primary	22%
Urinary tract infection	15%
Abdominal infection	14%
Respiratory	15%
Outcomes	
ICU admission	15%
Mechanical ventilation	10%
Septic shock	19%
Inappropiate empirical antibitic therapy	29%
Persistant BSI	13%
Mortality (30 days)	20%

**Table 2:** Logistic regression model of variables evaluated as predictive factors of carbapenem-resistant *Pseudomonas aeruginosa* bloodstream infections.

	OR (95% CI)	*p* Value
Age	0.98 (0.97–1.01)	0.151
Female	1.12 (0.71–1.77)	0.621
Solid organ transplant	1.39 (0.71–2.78)	0.343
Diabetes mellitus	1.15 (0.64–2.05)	0.634
COPD	0.77 (0.31–1.90)	0.584
HIV	1.54 (0.59–3.98)	0.372
Hematologic malignancy	2.71 (1.33–5.51)	0.006
Solid malignancy	0.89 (0.46–1.72)	0.743
Neutropenia	0.65 (0.33–1.29)	0.224
Hospital-acquired	0.73 (0.34–1.58)	0.429
Urinary catheter	0.76 (0.46–1.26)	0.292
Vascular catheter	1.12 (0.74–1.70)	0.569
Steroid therapy	0.79 (0.48–1.30)	0.362
Prior carbapenem therapy	2.37 (1.46–3.86)	<0.001
Source	1.34 (0.84–2.14)	0.217
Persistent bacteremia	0.60 (0.29–1.23)	0.168
Septic shock	1.00(0.57–1.76)	0.975

**Table 3:** Logistic regression model of variables evaluated as predictive factors of mortality in patients presenting with *Pseudomonas aeruginosa* bloodstream infections.

	OR (95% C.I.)	*p* Value
Sex	0.86 (0.60–1.23)	0.417
Age	1.03 (1.02–1.04)	<0.001
Solid organ transplant	0.58 (0.31–1.11)	0.104
Hospital-acquired	1.42 (0.83–2.43)	0.196
Diabetes mellitus	1.13 (0.73–1.75)	0.560
COPD HIV Hematologic malignancy Solid malignancy	1.01 (0.59–1.70) 1.88 (0.78–4.55) 1.87 (1.11–3.15) 1.11 (0.70–1.74)	0.970 0.157 0.017 0.645
Neutropenia	0.88 (0.53–1.47)	0.640
Urinary catheter	2.05 (0.37–3.06)	<0.001
Steroid therapy	1.35 (0.92–1.98)	0.114
Persistent bacteremia	1.64 (0.97–2.77)	0.065
Septic shock	6.57 (4.54–9.51)	<0.001
High-risk source *	4.96 (3.32–7.43)	<0.001
Adequate empirical therapy	0.52 (0.35–0.75)	0. 001
Carbapenem-resistant PAE	1.53(1.01–2.29)	0.036

* All sources except urinary tract infection and catheter-related infection.

**Table 4:** Demographics and clinical characteristics of the cases with *P. aeruginosa* BSI and their outcomes in solid organ transplant recipients.

	Total n = 2057	SOT Patients n = 265	Non-SOT Patients n = 1792	*p*
Men	64%	76%	63%	<0.001
Age, median (SD)	63 (SD19)	56 y (SD 13)	65 y (SD 20)	0.03
Heart transplant		9 (3%)		
Kidney transplant		130 (549%)		
Liver transplant		105 (40%)		
Kidney–pancreas transplant		21 (8%)		
Comorbidities				
Diabetes mellitus	18%	24%	17%	0.008
COPD *	10%	5%	11%	0.006
Ischemic cardiopathy	15%	11%	15%	0.07
Hematologic malignancy	22%	5%	25%	<0.001
Liver cirrhosis	6%	7%	6%	0.9
HIV	6%	1%	6%	<0.001
Risk factors				
Urinary catheter	33%	16%	33%	<0.001
Venous catheter	64%	68%	63%	0.28
Neutropenia	15%	8%	16%	0.003
Hospital-acquired	85%	92%	84%	0.04
Community-acquired	15%	8.3%	16.1%	0.001
Source				
Catheter-related	31%	25%	32%	<0.001
Primary	22%	16%	23%	0.8
Urinary tract infection	15%	27%	13%	0.02
Abdominal infection	14%	14%	14%	0.9
Respiratory	15%	11%	16%	0.04
Outcomes				
ICU admission	15%	11%	16%	0.002
Mechanical ventilation	10%	7%	11%	0.034
Septic shock	19%	18%	19%	0.85
Inappropiate empirical antibitic therapy	29%	28%	29%	0.34
Persistant BSI	13%	9%	14%	0.3
Mortality (30 days)	20%	13%	21%	0.002

* COPD: chronic obstructive pulmonary disorder.

**Table 5:** Logistic regression model of variables evaluated as predictive factors of carbapenem-resistant *Pseudomonas aeruginosa* bloodstream infection in solid organ transplant recipients.

	OR (95% CI)	*p* Value
Age	0.98 (0.96–1.01)	0.322
Female sex	1.12 (0.54–2.33)	0.745
Diabetes mellitus	0.62 (0.29–1.29)	0.848
COPD	3.27 (0.71–14.9)	0.127
HIV	2.45 (0.10–55.2)	0.571
Hematologic malignancy	1.45 (0.21–9.84)	0.698
Solid malignancy	0.87 (0.21–3.49)	0.848
Neutropenia	1.18 (0.31–4.50)	0.806
Nosocomial acquisition	1.72 (0.48–6.19)	0.401
Urinary catheter	1.98 (1.1–3.69)	0.031
Venous catheter	2.05 (1.8–4.25)	0.013
Steroid therapy	0.82 (0.38–1.76)	0.616
Prior carbapenem therapy	4.49 (2.10–9.95)	<0.001
Source	1.01 (0.99–1.08)	0.659
Persistent bacteremia	2.23 (0.81–6.19)	0.120
Shock	0.67 (0.28–1.58)	0.366

## Data Availability

Data supporting reported results can be delivered upon reasonable request.
